# A Ternary Catalytic System for the Room Temperature Suzuki-Miyaura Reaction in Water

**DOI:** 10.1155/2013/456789

**Published:** 2013-10-30

**Authors:** Aires da Conceição Silva, Jaqueline Dias Senra, Andréa Luzia Ferreira de Souza, Luiz Fernando Brum Malta

**Affiliations:** ^1^Instituto de Química, Universidade Federal do Rio de Janeiro, CT Bloco A Lab 641, 21941-909 Rio de Janeiro, RJ, Brazil; ^2^Núcleo de Pesquisas de Produtos Naturais, Universidade Federal do Rio de Janeiro, CCS Bloco H, 21941-614 Rio de Janeiro, RJ, Brazil; ^3^Instituto de Química, Universidade Federal do Rio de Janeiro, 27930-560 Macaé, RJ, Brazil

## Abstract

The formation of Pd(0) in the absence of any classical reducing agent in a medium containing Mg^2+^/Al^3+^ layered double hydroxide (LDH) and *N,N*-dimethylformamide was evidenced. XRD analysis showed the presence of crystalline phases of palladium in the Pd/LDH composite. Suzuki-Miyaura reactions in aqueous medium were carried out at room temperature, and good yields were obtained with bromoarenes and iodoarenes using the ternary system LDH-Pd-CD (cyclodextrin) as catalyst.

## 1. Introduction

 Suzuki-Miyaura reaction is one of the most useful palladium-catalyzed carbon-carbon bond-forming coupling reactions which involve organic electrophiles and organoboron compounds [1–3]. In general, the easy availability of the starting materials (organic halides and boronic acids), high reactivity under mild reaction conditions, the tolerance of a wide range of functionalities, the formation of nontoxic products, the small amount of catalyst used in the reaction, and the possibility of using water as a solvent or cosolvent contribute to their increasing interest [4]. Focus on sustainable development has directed numerous environmental planning of new synthetic methodologies [5]. In this perspective, catalytic processes that minimize or avoid the use of toxic agents are highly desirable. In recent years, the use of soluble supramolecular receptors such as cyclodextrins, has excelled in the most efficient approaches in catalysis taking into account the focus on environmental issues [6].

 Cyclodextrins (CDs), cyclic oligosaccharides obtained from the enzymatic degradation of starch, have been applied in several important synthetic transformations [7]. Structurally, they are composed of residues of D-glucopyranose attached to *α*-1,4-linkages. Due to their hydrophobic cavity, a large number of organic compounds can form inclusion complexes of host-guest type in aqueous medium, which causes significant increase of solubility for guest molecules. CDs have been used in catalysis [7, 8], in systems of controlled release of drugs [9, 10], and in selective removal of organic substrates in wastewater contaminated [11]. CDs can also act as reducing agents and stabilizers of metal nanoparticles [12, 13]. 

Layered double hydroxides (LDHs), hydrotalcite-like compounds, are anionic clays since they present in their interlayer domains anionic species [14]. LDHs can be represented by the general formula [M_1−*x*_^II^M_*x*_^III^(OH)_2_]^*x*+^[A_*x*/*n*_^*n*−^·mH_2_O]^*x*−^, where M^II^ and M^III^ are divalent and trivalent cations, respectively; the value of *x* is equal to the molar ratio of M^III^/(M^II^+ M^III^), and A is the interlayer anion of valence *n* [15]. The structure of LDHs is based on the positively charged brucite-like sheets, and the positive charges are balanced by anions intercalated in the interlayer regions [16]. LDHs have many applications in heterogeneous catalysis as catalysts or catalyst precursors [17–20].

 We believe that the association among cyclodextrins, LDHs, and palladium could exert an outstanding performance towards the Suzuki reaction. Many reactants for this reaction are not soluble in water, and this can be remediated by using cyclodextrins and their phase transfer property. Additionally, excellent performances have been reported for the LDH-palladium composite in C–C cross-coupling reactions [18, 20]. Therefore, our work shed light on the influence of the ternary system LDH-Pd-CD on the Suzuki reaction under mild conditions applying aqueous medium.

## 2. Experimental

### 2.1. Materials

 All chemicals were of reagent grade or analytical grade when available and were used without further purification. All aqueous solutions were prepared with Milli-Q water.

### 2.2. Synthesis of LDH

The Mg/Al layered double hydroxide was obtained using a solution containing 0.006 mol of Mg(NO_3_)_2_·6H_2_O (Vetec, 99%) and 0.003 mol of Al(NO_3_)_3_·9H_2_O (Vetec, 99%) (Mg(II)/Al(III) = 2) in 100 mL of Milli-Q water. Under vigorous stirring, LDH was prepared by coprecipitation at pH = 10 with a 1.0 mol·L^−1^ solution of NaOH (Vetec, 99%) at room temperature. The suspension thus obtained was filtered, washed with Milli-Q water, and dried over a stove. The LDH was obtained as a white solid.

### 2.3. Synthesis of Pd/LDH Composite

 Pd/LDH composite was prepared by mixing 0.4 g LDH with a 0.025 mol·L^−1^ Na_2_PdCl_4_ (Aldrich, 98%) solution in *N,N*-dimethylformamide (Aldrich, 99.8%) (Pd : LDH; 1 : 2) at 80°C for 24 h. Initially, the palladium solution was yellow; some minutes after LDH addition to the system, the mixture started to darken, and after about 1 hour of reaction, a black colored precipitate appeared. After 24 hours of reaction, the entire solution became dark. The black solid obtained was filtered, washed with portions of *N,N*-dimethylformamide (DMF), and dried in a properly stove.

### 2.4. General Procedure for Suzuki Reaction

In a 25 mL reaction flask the aryl halide (1.0 equivalent), 1.2 equivalents of phenylboronic acid (Aldrich, 95%), 2.0 equivalents of K_2_CO_3_ (Aldrich, 99.9%), the palladium catalyst, and 5 mL of the solvent were added at room temperature. After the end of reaction, the reaction mixture was extracted with 20 mL of diethyl ether (Vetec, 99.5%). The organic phase was then filtered on Celite, washed with water, and dried under anhydrous Na_2_SO_4_ (Aldrich, 99%). The solution was filtered and the solvent was evaporated. The product thus obtained was characterized by GC-MS and ^1^H-NMR.

### 2.5. Materials Characterization

 Powder X-ray diffraction (XRD) patterns were recorded on a Rigaku Ultima IV diffractometer using Cu K*α* radiation. Scans were performed over 2*θ* range from 5° to 80°, using a resolution of 0.05° and count time of 1 s at each point.

 Fourier-transform infrared (FTIR) spectra were recorded on a Nicolet Magna-IR 760 spectrophotometer with resolution of 4 cm^−1^ and a number of 16 scans using wavenumber range from 400 to 4000 cm^−1^. Samples were prepared by mixing the powdered solids with KBr.

 X-ray fluorescence (XRF) analysis was performed on a Philips PW 2400 sequential wavelength X-ray spectrometer.

 Scanning electron microscopy (SEM) and energy-disperse X-ray spectroscopy were performed on a JEOL JSM 6460-LV microscope operating among 10–20 kV and equipped with an energy-disperse X-ray spectrometer.

 BET surface areas, pore diameters, and pore volumes were calculated from nitrogen adsorption-desorption isotherms that were obtained at 77.3 K using a Quantachrome NovaWin instrument. Samples were pretreated under vacuum at 453 K for 5 h prior to use. Specific surface areas were calculated by the BET method [21], and pore distribution was established by the BJH method [22].

The analysis of gas chromatography-mass spectrometry (GC-MS) was performed on a chromatograph model GCMS-QP2010 Plus with a RTX-5MS column (crossband, with the stationary phase of 5% diphenyl and 95% dimethyl polysiloxane) about 30 m × 0.25 mm × 0.25 *μ*m thick. The carrier gas used was He (100.0 kPa) at a flow rate of 1.33 mL/min. The GC interface with a mass selective detector was maintained at 240°C. The program used in the analysis was as follows: injector temperature = 240°C, detector temperature = 260°C, column temperature = 100°C–240°C, the rate of change of column temperature = 10°C/min, and injection volume = 0.5 *μ*L.

## 3. Results and Discussion

### 3.1. Powder X-Ray Diffraction

**Figure 1:**
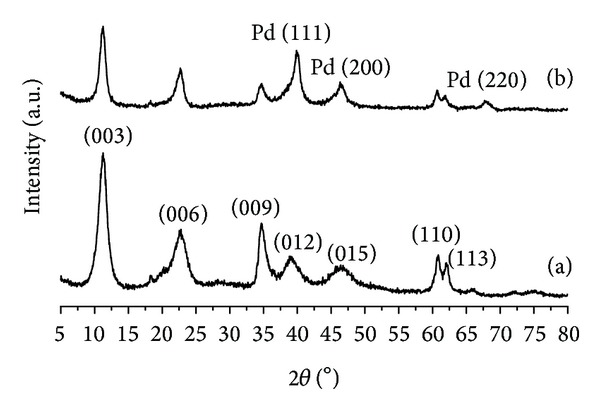
XRD patterns of (a) LDH and (b) Pd/LDH composite.

Figure 1 shows the XRD patterns of LDH (a) and Pd/LDH composite (b). The LDH pattern (Figure 1(a)) is typical of a hydrotalcite-like layered material, having been indexed according to the American Mineralogist card no. 0014738.

The XRD pattern of Pd/LDH composite (Figure 1(b)) showed three additional peaks (Figure 1(a)) at 2*θ* = 40.15°, 46.7°, and 68.15°. Past works in the literature have reported some modifications in the LDH diffraction pattern supposedly due to the PdCl_4_^2−^ intercalation [18]. In the present work, we searched for the source of the changes observed in the XRD pattern of Pd/LDH in face of that of LDH. Based on Crystmet card #AL3277 we identified these additional reflections as corresponding to the three planes (111), (200), and (220) of elemental palladium phase that somehow was formed in the material [23, 24].

### 3.2. Fourier-Transform Infrared Spectroscopy

**Figure 2:**
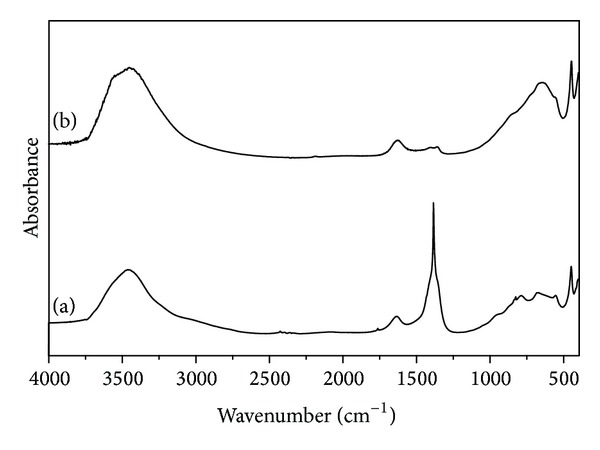
FTIR spectra for (a) LDH and (b) Pd/LDH composite.

Figure 2 shows the FTIR spectra for LDH and for Pd/LDH composite in the regions between 400 and 4000 cm^−1^. Both of them exhibit a broad band at 3500 cm^−1^ that can be assigned to O–H stretching, a band at 1630 cm^−1^ that is typical of the angular deformation vibration of the water molecule, and a band at 450 cm^−1^ that can be assigned to the Al–OH vibrations. Concerning the strong band centered at 1384 cm^−1^ for LDH it can be assigned as the asymmetric stretching of nitrate (NO_3_^−^). Since this signal almost disappears in the spectrum of Pd/LDH composite (Figure 2(b)), it may be inferred that the ion-exchange process occurred, corroborating the CHN elemental analysis (Table 1). This analysis indicated a decrease in the amount of nitrogen (from NO_3_^−^) for Pd/LDH in face of LDH. Another important feature to be discussed in the Pd/LDH composite spectrum is the increase of intensity for the CO_3_^2−^ in-plane bending mode at 642 cm^−1^ which according to the literature [25] is correlated with the intercalation process of PdCl_4_^2−^ in LDH.

### 3.3. Scanning Electron Microcopy and X-Ray Energy Dispersive Spectroscopy

**Figure 3:**
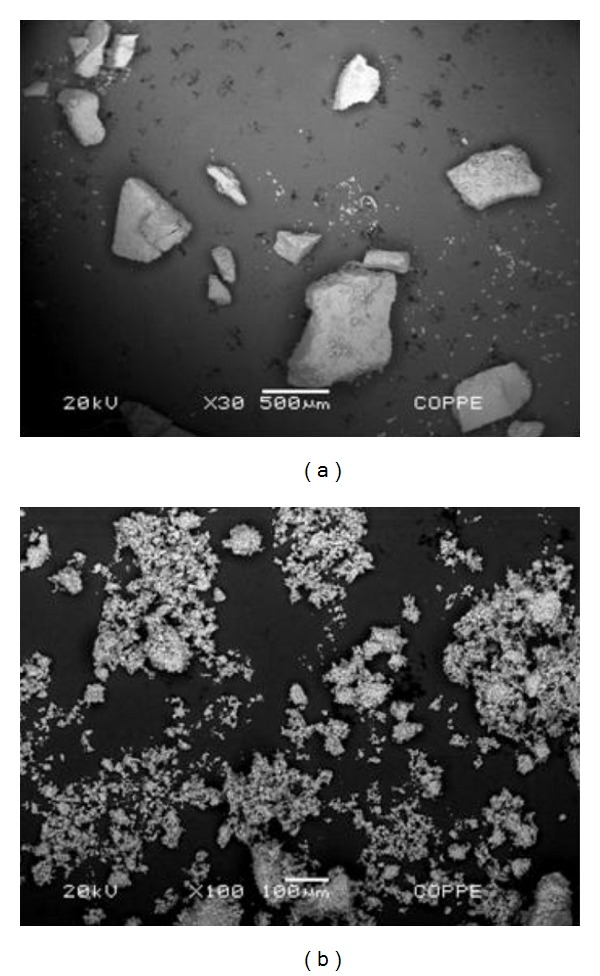
SEM images of LDH ((a) magnification: 30x) and Pd/LDH composite ((b) magnification: 100x).

As part of the morphological analysis, the scanning electron microscopy images from LDH and Pd/LDH composite employing backscattered electrons signals are shown in Figure 3. The left image in Figure 3 evidences that LDH presents large and heterogeneously sized agglomerates while the right image shows that Pd/LDH composite is more dispersed and homogeneous, with smaller particles.

**Figure 4:**
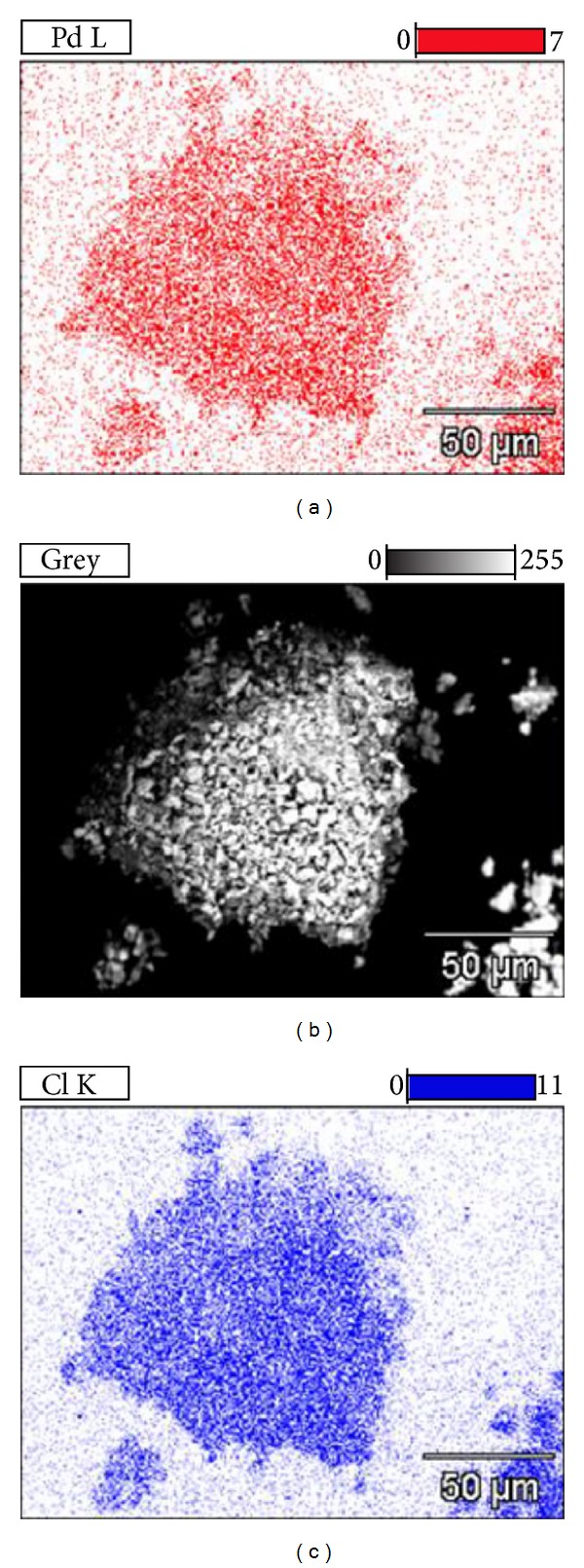
Images of characteristic X-ray emitted by Pd and Cl elements in the Pd/LDH composite.

The images of emitted X-rays (Figure 4) for the Pd/LDH composite obtained via X-ray energy dispersive spectroscopy show homogeneous distribution of palladium (in red) and chlorine (in blue) throughout the sample. This observation supports that there is no second phase containing palladium segregated from the matrix for this composite, which permits to infer that the metal was inserted in the interlayer space.

The analysis by X-ray fluorescence allowed quantitatively establishing the percentage that each element has in the Pd/LDH composite: Pd: 1.06%; Mg: 20.13%; Al: 11.85%.

### 3.4. Textural Properties

Table 2 lists the specific surface areas, average pore diameters, and pore volumes of LDH and Pd/LDH composite. Pd/LDH composite has greater specific surface area than LDH due to the empty spaces generated in the interlayer region that arises from the stoichiometry of ion-exchange process, two NO_3_^−^ by one PdCl_4_^2−^. 

**Scheme 1:**
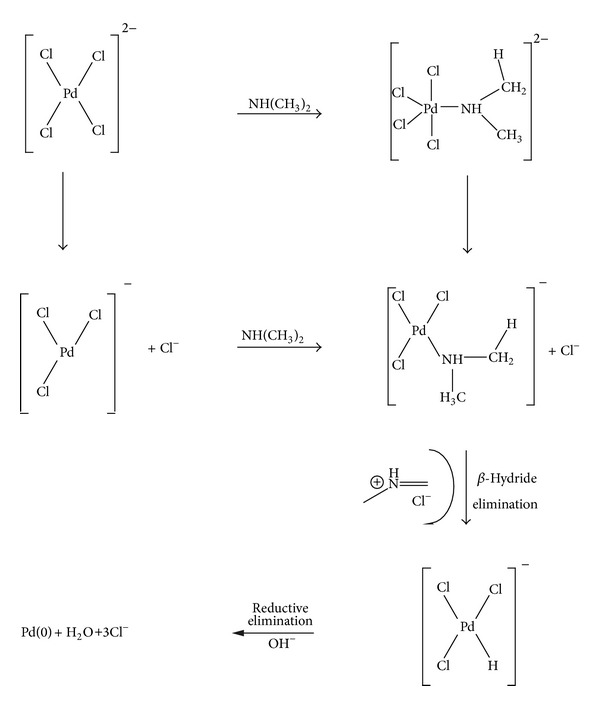
Mechanistic proposal for the dimethylamine-mediated reduction of Pd(II) in the interlayer space.

With all this in mind it is evident that in a first moment the ion-exchange process occurs between NO_3_^−^ and PdCl_4_^2−^ species followed by a second event in which Pd(II) is reduced to Pd(0). Concerning the latter event, we are not aware of any means by which LDH matrix could exert the metallic reduction process. However it is important to say that the ion-exchange process was carried out in the presence of *N,N*-dimethylformamide. It is common sense that DMF is not stable in the presence of strong acids or bases. Talking about hydroxides, the products of DMF decomposition are formate ion and dimethylamine by alkaline hydrolysis [26]. By the mechanistic proposal presented in Scheme 1 the formation of palladium (0) is explained from the oxidation of dimethylamine to the corresponding iminium ion. We proposed two mechanisms (associative or dissociative pathways) concerning the substitution of chloride ion by dimethylamine. On the last step the reductive elimination aided by OH^−^ species leads to the formation of Pd(0).

### 3.5. Suzuki Reactions

 Due to the importance of the Suzuki reactions, we proposed a new method for them. Firstly we tested our catalytic system with the model reaction between 4-bromoacetophenone and phenylboronic acid. All the results are summarized in Table 3.

 Experiments to obtain the best solvent composition were processed according to the conditions described in Table 3, entries 1–4. We chose Na_2_PdCl_4_ 1% due to the use of its anion as intercalated species in LDH matrices. Since it is our aim to perform reactions in water-based medium, all solvent compositions contain water in a major percentage. Beyond water, isopropyl alcohol was also tested since it can act as a reducing agent of Pd(II) (unpublished results). 

The result from entry 1, reaction in neat water, shows that the system is hardly reactive in a time of 24 hours at room temperature, with a conversion of 26%. In entries 2–4 solvent mixtures between water and isopropyl alcohol were used in different proportions (60–90% of H_2_O). In entries 2 and 3, applying proportions of water: isopropyl alcohol of 60 : 40 and 80 : 20, respectively, complete conversions of the aryl halide into the product were obtained. However the increase of the water content to 90%, entry 4, led to the conversion dropping to 70%, hence evidencing the influence of isopropyl alcohol in the solvent composition. This result was the starting point to investigate if the addition of cyclodextrin to the reaction medium could improve the conversion.

 The native *β*-cyclodextrin was used in the experiments 5–7 described in Table 3. Comparing entries 4 and 5 it is clear that the Pd : CD ratio of 1 : 1 in the reaction medium practically did not affect the conversion of the starting material. Therefore, a new reaction (entry 6) was carried out applying a Pd : CD ratio of 1 : 10. For this run an increase of conversion was observed, probably due to the presence of a greater amount of CD in the medium that can afford a greater dispersion of the reactants, a process that was benefited from the host (CD)-guest (organic molecule) interaction. When a Pd : CD ratio of 10 : 1 was tested (entry 7), a decrease in the conversion of 4-bromoacetophenone occurred, showing that there is an optimum ratio of Pd : *β*-CD (1 : 10), corresponding to entry 6.

 The search for the best cyclodextrin form to perform the aqueous Suzuki reaction took place in the next set of reactions. For the reaction of entry 8 the hydroxypropylated form of *β*-CD (HP-*β*-CD) was tested and an outstanding conversion of over 99% was achieved. Aiming to test the effect of an anionic cyclodextrin, the sodium salt of sulfated *β*-CD was used in the reaction of entry 9 and 86% of the halide was converted into products; however the reaction selectivity was not good, considering that the reacted mixture was 14% in biphenyl and 72% in 4-phenylacetophenone. In all other reactions, homocoupling products were negligible. Therefore, the use of this CD was discarded for future reactions. From this set of experiments, we chose HP-*β*-CD as additive for further reactions. 

The excellent result employing HP-*β*-CD in the medium of water : isopropyl alcohol (90 : 10) encouraged us to perform the same reaction in neat water. These experiments are related in Table 3, entries 10–12. The reaction in entry 10, using 1 mol% of the catalyst and a Pd : HP-*β*-CD ration of 1 : 10, gave conversion of over than 99% evidencing that isopropyl alcohol is not necessary to obtain a high product yield. With this result in mind, the following experiments took place in an attempt to reduce the amount of the catalyst in the reaction medium. Entry 11 shows a reaction where the amount of palladium was reduced by half (0.5%), but the conversion of the aryl halide was also significantly reduced. In an attempt to improve this result the reaction of entry 12 was performed using the same catalyst amount but applying a Pd : HP-*β*-CD ratio of 1 : 20, which afforded an excellent conversion of over than 99%.

 The time duration of the Suzuki reaction was screened in order to determine whether applying 24 hours for this reaction is really necessary. The experiments are related in Table 3, entries 13–17. These experiments proved that the Suzuki reaction occurred with a conversion of over than 99% after a period of only 8 hours (entry 17) at room temperature using a 1 : 20 ratio of Pd : HP-*β*-CD and 0.5% of homogeneous catalyst.

 In order to continuously improve our system and benefit from the advantages of heterogeneous catalysis, the Pd/LDH composite was used as the catalyst. The reaction in entry 18 shows that the heterogeneous catalyst converts over than 99% of the starting material into product. In entry 19, the reaction was carried out applying the Pd/LDH composite but in the absence of CD: the aryl halide conversion decreased to 93%. This result evidences that there is a synergism between the heterogeneous catalyst and the cyclodextrin. A third reaction (entry 20) was performed without the catalyst (palladium) but with the LDH matrix, and no conversion of the aryl halide into products has occurred.

The reaction of entry 21 was an attempt to reduce the amount of heterogeneous catalyst used in the Suzuki reaction. Reducing it to 0.1% still permitted a conversion of the starting material greater than 99%; however further decrease of Pd amount to 0.01% led to a conversion of only 30%. Therefore the amount of Pd in the reaction was fixed at 0.1%. The run of entry 23 was an attempt to reduce the reaction time (3 hours) with the same amount of catalyst, but the conversion has also decreased (60%). Thus, the ideal condition found for the Suzuki reaction in aqueous medium was the one that has employed 0.1% of Pd/LDH composite at room temperature during 8 h with a Pd : HP-*β*-CD ratio of 1 : 20 (entry 21).

**Figure 5:**
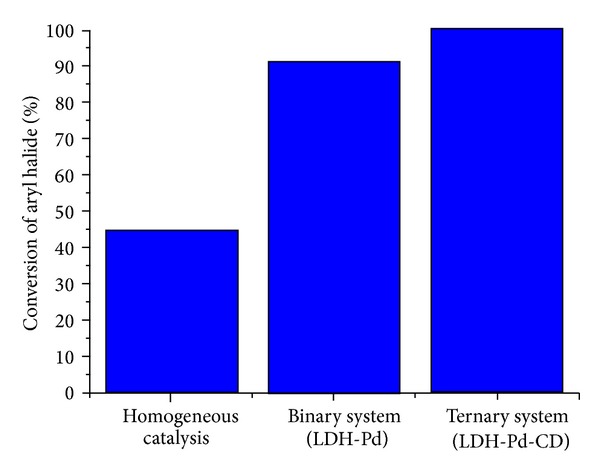
Differences among homogeneous catalysis, binary system (LDH-Pd) and ternary system (LDH-Pd-CD) in the reaction of iodobenzene and phenylboronic acid.

In order to verify the effectiveness of the ternary system LDH-Pd-CD, another substrate (iodobenzene) was used in the reaction, entries 24–26 of Table 3. The entry 24 corresponds to the reaction via homogeneous catalysis (Na_2_PdCl_4_ 0.1%) and shows a conversion of only 45% in the presence of HP-*β*-CD. The employment of LDH-Pd, with no CD in the reaction medium, improved the conversion to 91% (entry 26). However, the completion of the reaction was almost achieved when the ternary system LDH-Pd-CD was tested showing a conversion greater than 99% (entry 25).  Figure 5 represents entries 24–26.

The capacity of recycling the heterogeneous catalyst was investigated, and good yields were obtained up to two runs (1st run: 88%, 2nd run: 71%, and 3rd run: 55%).

 To verify the scope of our method employing the ternary system LDH-Pd-CD, other substrates were used (Table 4).

The reaction with 4-bromoacetophenone has provided a good yield of 4-phenylacetophenone due to the presence of an electron-withdrawing group (entry 1). For iodoarene substrates (entries 2–4) good yields were independently found whether electron-withdrawing (entry 3) or electron-donating groups (entry 4) are present. The reaction of bromobenzene (entry 5) provides a conversion of 93% to biphenyl, showing that the conversion is not as good as that of iodobenzene (entry 2). Entries 6 and 7 present the less reactive substrates (chloroarene substrates) affording low yields (<20%) even after 24 hours of reaction under reflux. The results of entries 2, 5, and 6 are in conformity with the order of reactivity of chloro-, bromo-, and iodoarenes towards the oxidative addition.

### 3.6. Spectral Data

*Biphenyl*. White solid. ^1^H NMR (CDCl_3_, 200 MHz) *δ* 7.81–7.65 (4H, d), 7.55–7.42 (4H, dd), 7.37–7.27 (2H, d); GC-MS: *m*/*z* = 77, 154.

*4-Nitrobiphenyl*. Yellow solid. ^1^H NMR (CDCl_3_, 200 MHz) *δ* 8.30 (2H, d), 7.74 (2H, d), 7.64 (2H, d), 7.52–7.44 (3H, m); GC-MS: *m*/*z* = 141, 152, 199.

*4-Phenylacetophenone*. Pale yellow solid. ^1^H NMR (200 MHz, CDCl_3_) *δ* 8.06–8.02 (2H, d), 7.72–7.62 (4H, m), 7.52–7.27 (3H, m), 2.65 (3H, s); GC-MS: *m*/*z* = 76, 152, 181, 196.

*4-Methylbiphenyl*. White solid. ^1^H NMR (200 MHz, CDCl_3_) *δ* 7.59 (2H, d), 7.49 (2H, d), 7.40 (2H, dd), 7.34 (1H, d), 7.28 (2H, d), 2.46 (3H, s); GC-MS: *m*/*z* = 152, 168.

## 4. Conclusion

 This work has evidenced the presence of Pd(0) for the Pd/LDH composite. It means that Pd (II) reduction occurred in the presence of layered double hydroxide and *N,N*-dimethylformamide, and to our knowledge it is the first time that such mechanism of reduction is suggested. The same proposal may apply to the formation of gold particles on LDH, recently studied by our group [27]. The ternary system LDH-Pd-CD at room temperature in aqueous medium has proven to be effective as a method for the Suzuki reaction employing iodo- and bromoarenes substrates. 

Taking into account the results obtained for the Suzuki reaction, the ternary catalytic system has potential to be successfully employed in other carbon-carbon cross-coupling reactions, as Heck, Sonogashira and Stille reactions.

## Figures and Tables

**Table 1:** CHN elemental analysis of LDH and Pd/LDH composite.

Sample	C (%)	H (%)	N (%)
LDH	1.25	3.73	3.56
Pd/LDH composite	1.29	3.71	0.21

**Table 2:** Textural properties of LDH and Pd/LDH composite.

Sample	*S*_BET_ (m^2^/g)^a^	*d*_*p*_ (nm)^b^	*V*_*p*_ (cm^3^/g)^c^
LDH	6.099	2.161	0.0169
Pd/LDH composite	7.834	1.736	0.0176

^a^Specific surface area. ^b^Pore diameter. ^c^Pore volume.

**Table 3:** Screening of the best condition for the Suzuki reaction between 4-bromoacetophenone and phenylboronic acid.

Entry	Solvent	Catalyst	Pd : CD	Cyclodextrin	Time (h)	Conversion^a^ (%)
1	H_2_O	Na_2_PdCl_4_ 1%	—	—	24	26
2	H_2_O : isopropyl alcohol (60% : 40%)	Na_2_PdCl_4_ 1%	—	—	24	>99
3	H_2_O : isopropyl alcohol (80% : 20%)	Na_2_PdCl_4_ 1%	—	—	24	>99
4	H_2_O : isopropyl alcohol (90% : 10%)	Na_2_PdCl_4_ 1%	—	—	24	70
5	H_2_O : isopropyl alcohol (90% : 10%)	Na_2_PdCl_4_ 1%	1 : 1	*β*-CD	24	68
6	H_2_O : isopropyl alcohol (90% : 10%)	Na_2_PdCl_4_ 1%	1 : 10	*β*-CD	24	86
7	H_2_O : isopropyl alcohol (90% : 10%)	Na_2_PdCl_4_ 1%	10 : 1	*β*-CD	24	59
8	H_2_O : isopropyl alcohol (90% : 10%)	Na_2_PdCl_4_ 1%	1 : 10	HP-*β*-CD	24	>99
9	H_2_O : isopropyl alcohol (90% : 10%)	Na_2_PdCl_4_ 1%	1 : 10	*β*-CD, sulfated sodium salt	24	86
10	H_2_O	Na_2_PdCl_4_ 1%	1 : 10	HP-*β*-CD	24	>99
11	H_2_O	Na_2_PdCl_4_ 0.5%	1 : 10	HP-*β*-CD	24	66
12	H_2_O	Na_2_PdCl_4_ 0.5%	1 : 20	HP-*β*-CD	24	>99
13	H_2_O	Na_2_PdCl_4_ 0.5%	1 : 20	HP-*β*-CD	2	57
14	H_2_O	Na_2_PdCl_4_ 0.5%	1 : 20	HP-*β*-CD	4	58
15	H_2_O	Na_2_PdCl_4_ 0.5%	1 : 20	HP-*β*-CD	6	89
16	H_2_O	Na_2_PdCl_4_ 0.5%	1 : 20	HP-*β*-CD	7	93
17	H_2_O	Na_2_PdCl_4_ 0.5%	1 : 20	HP-*β*-CD	8	>99
18	H_2_O	Pd/LDH 0.5%	1 : 20	HP-*β*-CD	8	>99
19	H_2_O	Pd/LDH 0.5%	—	—	8	93
20	H_2_O	HDL	—	HP-*β*-CD	8	—
21	H_2_O	Pd/LDH 0.1%	1 : 20	HP-*β*-CD	8	>99
22	H_2_O	Pd/LDH 0.01%	1 : 20	HP-*β*-CD	8	30
23	H_2_O	Pd/LDH 0.1%	1 : 20	HP-*β*-CD	3	60
24^b^	H_2_O	Na_2_PdCl_4_ 0.1%	1 : 20	HP-*β*-CD	8	45
25^b^	H_2_O	Pd/LDH 0.1%	1 : 20	HP-*β*-CD	8	>99
26^b^	H_2_O	Pd/LDH 0.1%	—	—	8	91

^a^Conversion of aryl halide into products measured by GC-MS.

^b^Reaction between iodobenzene and phenylboronic acid.

**Table 4:** Suzuki reactions between different aryl halides and phenylboronic acid using the ternary system (LDH-Pd-CD) at room temperature during 8 hours with 1 : 20 ratio of Pd : HP-*β*-CD.

Entry	Aryl halide	Product	Yield (%)
1	4-Bromoacetophenone	4-Phenylacetophenone	98^a^
2	Iodobenzene	Biphenyl	97^a^
3	4-Iodonitrobenzene	4-Nitrobiphenyl	98^a^
4	4-Iodotoluene	4-Methylbiphenyl	96^a^
5	Bromobenzene	Biphenyl	93^b^
6	Chlorobenzene	Biphenyl	13^b,c^
7	4-Chloronitrobenzene	4-Nitrobiphenyl	16^b,c^

^a^Isolated yield. ^b^Measured by GC-MS. ^c^24 hours under reflux.
